# Peer support and whole health coaching to address the healthcare needs of homeless veterans: a pilot study

**DOI:** 10.1186/s12875-022-01927-0

**Published:** 2022-12-19

**Authors:** Daniel Blonigen, David Smelson, Jennifer Smith, Nicole Baldwin, D. Keith McInnes, Ivan Raikov, Jillian Weber, Justeen Hyde

**Affiliations:** 1 Department of Veterans Affairs Palo Alto Health Care System, Health Services Research and Development Center for Innovation to Implementation, 795 Willow Road (152), Menlo Park, CA 94025 USA; 2 Department of Psychiatry and Behavioral Sciences, Stanford University School of Medicine, Stanford, CA USA; 3 HSR&D Center for Health Care Organization and Implementation Research, VA Bedford HealthCare System, Bedford, MA USA; 4 University of Massachusetts Chan Medical School, Worcester, MA USA; 5 Clinical Psychology Program, Palo Alto University, Palo Alto, USA; 6 Department of Health Law Policy and Management, Boston University School of Public Health, Boston, MA USA; 7 Department of Neurosurgery, Stanford University School of Medicine, Stanford, CA USA; 8 Veterans Health Administration, Homeless Program Office, Washington, DC USA

**Keywords:** Homelessness, Peers, Acute care, Primary care, Veterans

## Abstract

**Background:**

Homelessness is a robust social determinant of acute care service utilization among veterans. Although intensive outpatient programs have been developed for homeless veterans who are high utilizers of acute care (“super utilizers”), few scalable programs have been implemented to address their needs.

**Objective:**

Describe the development and pilot testing of a novel intervention that integrates the roles of a peer and whole health coach (“Peer-WHC”) in coordination with primary care teams to reduce homeless veterans’ frequent use of acute care.

**Design:**

Single-arm trial in three outpatient primary care clinics at a Veterans Health Administration (VHA) medical center; pre/post design using mixed-methods.

**Participants:**

Twenty veterans from VHA’s homeless registry who were super-utilizers of acute care and enrolled in primary care.

**Intervention:**

Weekly health coaching sessions with a peer over 12 weeks, including discussions of patients’ health care utilization patterns and coordination with primary care.

**Main measures:**

Rates of session attendance and intervention fidelity, patient-reported satisfaction and changes in patient engagement and perceptions of health, pre/post utilization of acute and supportive care services, and qualitative interviews with multiple stakeholders to identify barriers and facilitators to implementation.

**Key results:**

On average, patients attended 6.35 sessions (SD = 3.5, Median = 7). Satisfaction scores (M = 28.75 out of 32; SD = 2.79) exceeded a priori benchmarks. Patients’ perceptions of health improved from pre to post [*t*(df)=-2.26(14), *p* = 0.04]. In the 3-months pre/post, 45% (*n* = 9) and 15% (*n* = 3) of patients, respectively, were hospitalized. Qualitative feedback from patients, providers, and peers and fidelity metrics suggested value in increasing the length of the intervention to facilitate goal-setting with patients and coordination with primary care.

**Conclusion:**

Findings support the feasibility, acceptability, and utility of Peer-WHC to address the healthcare needs of homeless veterans. A future trial is warranted to test the impact of Peer-WHC on reducing these patients’ frequent use of acute care.

## Introduction

After steady declines from 2010 to 2016, the rate of homelessness among US adults is back on the rise [1]. This increase will likely translate to increased health problems in this population in the coming years as homelessness and health problems are inextricably linked [2]. For example, two-thirds of homeless adults in the US struggle with at least one chronic health condition, and the mortality rate among homeless adults is three times higher than housed individuals [3].

Homeless adults are disproportionately represented among acute care patients [4–6]. This is particularly true for the small number of high-need patients who account for half or more of acute care service costs [7]. These so-called “super-utilizers” have multiple, chronic health issues including substance use and mental health disorders and inadequate social support, all of which complicates their ability for self-care and engagement in supportive care services [7–9]. Homelessness is also a robust social determinant of super utilization of acute care in the veteran population [4–6], which are overrepresented among homeless adults in the US [10]. This overrepresentation may be due to higher rates of mental illness, such as posttraumatic stress disorder and substance use disorder, among veterans than non-veterans [3]. Further, many new episodes of homelessness among veterans occurs within 3 years of discharge from active duty [11]. Thus, the challenges associated with reintegrating back to civilian life, such as finding stable employment and housing, may account in part for the overrepresentation of veterans in the US homeless population.

To reduce acute care utilization for super-utilizers, intensive management approaches within primary-care, interdisciplinary teams have been recommended [12, 13]. In the Veterans Health Administration (VHA), such care is provided through Patient Aligned Care Teams (PACT) [14]. However, the evidence for the effectiveness of intensive outpatient programs to reduce acute care utilization among super-utilizers has been mixed [13, 15] and few scalable programs have been implemented for homeless adults per se. For example, relative to generalized PACTs, a specialized PACT for homeless veterans (“Homeless PACT”) has been found to reduce hospitalizations [16, 17]. However, most homeless veterans in VHA primary care are on traditional PACTs [18]. Consequently, approaches for improving the care and outcomes of the larger population of homeless veterans treated in traditional PACTs are needed. Interventions that have shown promise in this regard use peers to build trust with patients and reduce self-stigma [19–21] and incorporate patients’ preferences in treatment planning [19, 22].

In VHA, *peer specialists* are veterans with a history of substance use, mental illness, and/or homelessness who are now in recovery and trained to provide services to patients who are currently struggling with such issues. Peers are a high-value workforce that facilitate veterans’ engagement in supportive services [23]. Given an increasing need for mental health care since the onset of COVID-19 and the decreased availability of mental health providers, peers can provide the time and attention that is required for many vulnerable patient populations. In a prior RCT, homeless veterans on PACTs were randomized to receive (or not receive) peer mentoring. Although patient satisfaction with peer support was high [21], there were no between-group differences on acute care utilization or overall costs [24]. Importantly, the peer role in this study lacked a standardized curriculum to guide the patient-peer interactions.

One standardized approach that capitalizes on key facilitators of engagement in supportive services among homeless veterans is *Whole Health Coaching *[25]*.* This approach is guided by patients’ values and goals, rather than treatment of specific conditions, and entails a holistic approach to patient needs across a number of areas of self-care. Health coaches help patients develop a personalized health plan oriented to their personal health goals and priorities for self- and professional care. This approach reduces self-stigma and increases patient activation, which can increase engagement in supportive services and, in turn, reduce reliance on acute care services [25].

In this paper, we describe the development of a novel intervention (*Peer-Supported Whole Health Coaching*; “Peer-WHC”) to address the healthcare needs of homeless super-utilizers. This approach integrates the roles of a peer specialist and health coach who works in coordination with a patient’s PACT. The goal of this intervention is to address a patient’s personal health goals via linkage and engagement in supportive care services and ultimately reduce patients’ use of acute care services. We chose to develop and pilot this intervention because existing services for homeless veterans focus on clinical and housing stabilization but do not systematically include a focus on development of personal health goals that are aligned with patients’ priorities and values. Thus, Whole Health Coaching is a key innovation to be added to existing services for homeless veterans. We evaluated the feasibility, acceptability, and utility of this approach among homeless super-utilizer veterans, which included collection of qualitative data to identify facilitators and barriers to patient engagement and satisfaction with the intervention and its implementation in primary care.

## Methods

### Sample and procedures

Patients were recruited from one of three community-based outpatient clinics at a VHA medical center. Patients were identified from reports generated through the “Hot Spotter” dashboard on VHA’s Support Services Center, which flags patients who are (a) on VHA’s Homeless Registry–i.e., those who utilized any VHA homeless services in the past two years, (b) enrolled on a PACT, and (c) had a hot spotter event in each of the past two quarters of the fiscal year. A hot spotter event is defined as ≥ 1 hospitalization or ≥ 2 Emergency Department [ED] visits in a fiscal quarter. RAs reviewed these reports weekly from January 2020–March 2021, which identified an initial pool 152 patients. Using chart reviews, 51 patients (33.6%) were excluded due being flagged as high-risk for suicide or violence (*n* = 16; these patients were not referred for additional care as they were already engaged in supportive care services in the VHA), living more than a 1-hour drive from their assigned outpatient clinic (*n* = 28), hospitalized long-term (*n* = 5), or deceased (*n* = 2). A letter was mailed to the remaining 101 eligible patients. Patients who did not respond to the letter were called one week later. Among eligible patients, 54 (53.5%) responded to the letter or were able to be reached by phone, 25 (46.3%) of whom expressed interest in participating. Twenty patients were enrolled (five were wait-listed).

Regarding sample characteristics (see Table 1), participants were all male, mostly White (75%) with 25% reporting their ethnicity as Hispanic/latinx. On average, participants were 54.9 years old (SD = 14.6), had 13 years of education (SD = 1.34), and were homeless for 10.4 months (SD = 11.8) in the past three years. Most participants (90%) reported their current housing situation as stable. The number of participants who were either married/cohabitating, widowed/separated/divorced, or never married was nearly equal. Half of participants were disabled/retired. Based on ICD-10 codes from their VA service utilization records in the three months pre/post study enrollment, most participants (85%) had a diagnosis of a mental health disorder (mostly commonly posttraumatic stress disorder or a mood disorder) and another chronic medical condition (80%).

**Table 1:** Sample characteristics

Variable	N (%) or M (SD)
*Gender* (% male)	20 (100%)
*Race/Ethnicity*	
White (Non-Hispanic)	10 (50%)
Hispanic	5 (25%)
Black/African American	2 (10%)
Asian	2 (10%)
American Indian	1 (5%)
*Age* (M, SD)	54.9 (14.6)
*Years of Education* (M, SD)	13 (1.34)
*Housing Status*	
Currently Stably Housed	18 (90%)
Months of Homelessness (past 3 years) (M, SD)	10.4 (11.8)
*Marital status*	
Married/Co-Habitating	6 (30%)
Never married	7 (35%)
Widowed/Separated/Divorced	7 (35%)
*Employment Pattern (past 3 years)*	
Disabled/Retired	10 (50%)
Unemployed	5 (25%)
Employed	5 (25%)
*Total Income Per Month* (M, SD)	$2,189 ($1,691)
*Diagnoses*	
Any mental health condition	17 (85%)
Substance use disorder	7 (35%)
Posttraumatic stress disorder	11 (55%)
Mood disorder	12 (60%)
Anxiety disorder	5 (25%)
Serious mental illness	3 (15%)
Other mental health condition	3 (15%)
Other chronic medical condition	16 (80%)

Participants completed a baseline interview by phone to obtain information on sociodemographics, patient engagement, and perceptions of health. At the end of the interview, participants were provided with the name of a peer who would contact them by phone the next day. After three months, patients were reinterviewed by phone (*n* = 16; 80% retention) to measure changes in patient engagement and perceptions of health, and to obtain feedback on their perceptions of the intervention and overall satisfaction. Participants were paid $50 for each interview. Service utilization data in the three months pre/post enrollment were obtained from VA’s Corporate Data Warehouse. Study procedures were approved by the local institutional review board.

VA staff who were care providers of participants and leaders of the outpatient clinics were also contacted by research staff to participate in a one-time semi-structured interview (30-minutes long; audio-recorded). Seventeen individuals were contacted, 6 (35.3%) of whom agreed to participate (4 providers; 2 clinic leaders). Participant positions included 1 primary care physician, 1 nurse practitioner, 1 nurse care manager 1 occupational therapist, 1 homeless service case manager, and 1 social worker.

### Peer-supported Whole Health Coaching

**Fig. 1:**
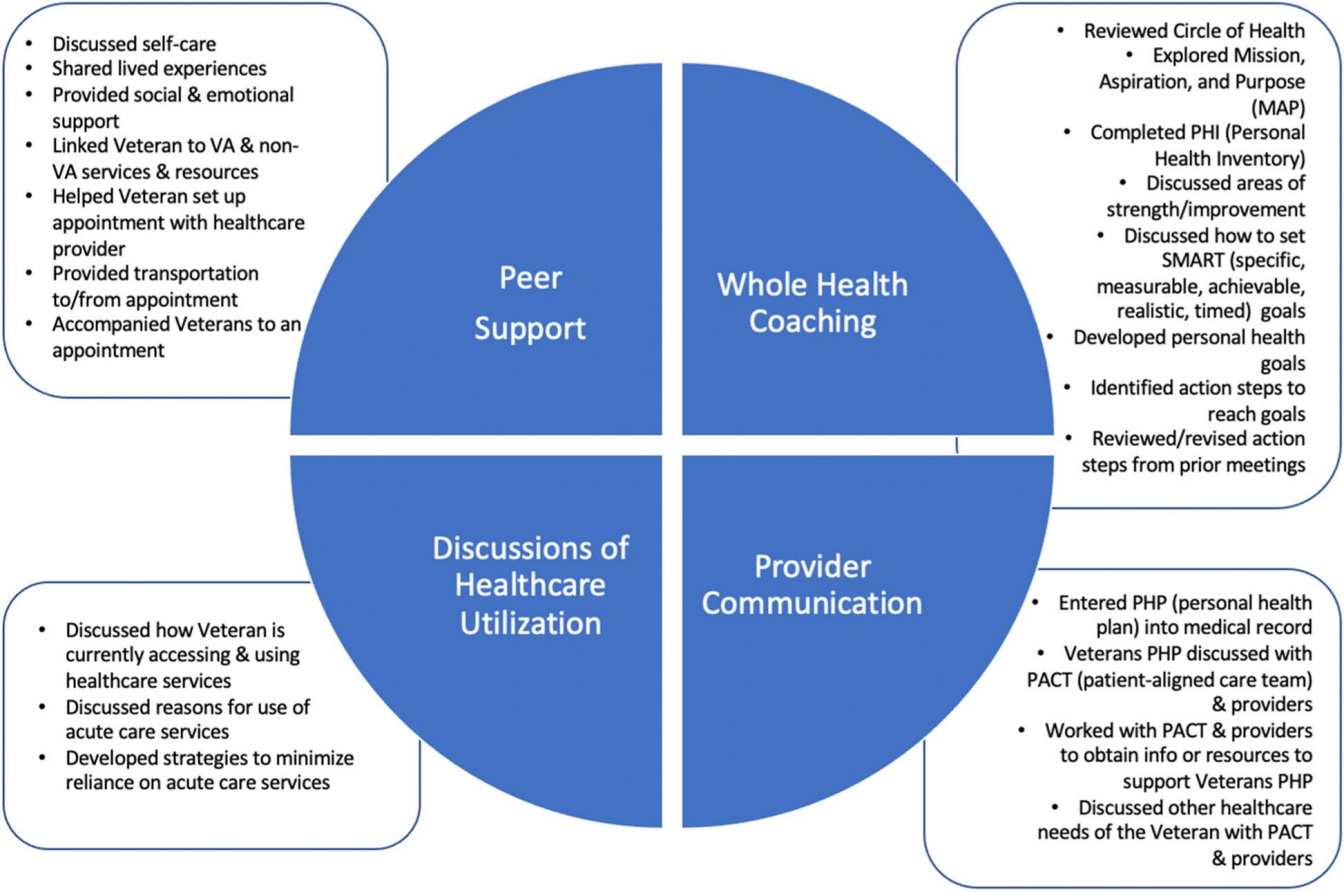
Major components and activities of the Peer-Supported Whole Health Coaching model

Participants met weekly with the same peer over 12 weeks. Sessions were scheduled for 30–60 min with one of two peers, both of whom had lived experience with homelessness and were in recovery from substance use and/or mental health problems. Sessions were offered in-person or by phone or video. The intervention comprised four categories of activities on the part of the peer (for additional details, see Fig. 1):

- *Peer Support*: Peers engaged as needed in the core activities of a Certified Peer Specialist [26] – i.e., sharing lived experiences of homelessness and/or recovery from substance use and mental health problems, discussing self-care, providing social/emotional support, linking patients to VHA and non-VHA services and resources, helping patients set up appointments with providers, providing transportation to and from appointments, and accompanying patients to appointments.
- *Whole Health Coaching*: Peers introduced patients to the Circle of Health, which comprises 8 interconnected areas of self-care (Working Your Body; Surroundings; Personal Development; Food & Drink; Recharge; Family, Friends, & Coworkers; Spirit & Soul; and Power of the Mind). Next, peers explored patients’ mission, aspiration, and purpose, which entails learning about a patient’s interest and priorities to get them to reflect on what brings joy and meaning to their lives. Peers also assisted patients with completing a Personal Health Inventory, which included ratings of “where they are now” and “where they want to be” on each domain of the Circle of Health, and discussed patients’ personal strengths and areas of growth. Thereafter, peers reviewed how to set SMART goals, assisted patients with developing personal health goals, both in the short-term (e.g., next few weeks) and long-term (e.g., over 3–12 months); identified action steps to reach personal health goals; and reviewed action steps from prior meetings and revised the personal health plan as needed.
- *Discussions of healthcare utilization*: Prior to the first session, peers reviewed patients’ medical records to understand their reasons for using acute care services and identified potential barriers to access of supportive care services. Peers used this information to ask open-ended questions to learn about how patients were currently accessing and using healthcare services and their reasons for using acute care. (e.g., “What are your main types of care? What about your care works [doesn’t work] for you? What are your barriers to getting the care you need? What do you see as reasons for using the ED?”). Discussions were framed as wanting to learn how patients’ use of healthcare services matches their personal health goals, identify strategies to minimize reliance on acute care and improve their overall quality of care (e.g., providing coaching on how to have conversations with providers to talk about personal health goals; self-care strategies).
- *Provider communications*: In addition to patient sessions, peers communicated with patients’ VHA providers to share what they have learned from the patient in terms of what matters most to them and their personal health goals. To this end, peers completed a Personal Health Plan for the patient based on the patients’ ratings from the Personal Health Inventory and entered a note into patients’ medical records and included PACT providers as co-signers. Thereafter, peers contacted providers synchronously (e.g., attending team meetings) or asynchronously (e.g., emails; instant messages) to discuss patients’ Personal Health Plans. Peers also worked with PACT and other VHA providers to obtain information or resources to support patients’ Personal Health Plans.

To receive training in whole health coaching, peers attended a 6-day virtual training accredited by the National Board for Health & Wellness Coaching, which was divided into two 3-day sessions one month apart, with practice coaching in between [27]. Competency was evaluated by course trainers using a Coaching Skills Checklist. Prior to recruitment, peers also participated in a half-day long training led by a clinical psychologist (DB) and health coaching expert (JH) to review the study procedures and intervention components, which were detailed in a manual developed by these authors. Peers used VHA’s REDCap system to document patient sessions, including the mode of communication (in-person/phone/video), amount of time (in minutes) of direct contact with patients, and intervention activities that had engaged in. The REDCap records were used to index intervention fidelity. Clinical supervision was provided through weekly, one-hour long video meetings between the peers and two study authors (DB, JH).

### Measures

*Patient engagement* in their health and healthcare was measured pre/post with an 8-item version of the Altarum Consumer Engagement measure [28]. Items were rated on a 5-point scale (1 = Strongly Disagree, 5 = Strongly Agree) and summed (higher scores indicate greater levels of patient engagement). Scores were aggregated to compute a total score at each time point (α_Baseline_ = 0.49, α_3−months_ = 0.48) with higher scores indicating greater levels of patient engagement. *Perceptions of health* (e.g., physical, mental, social, overall quality of life) was measured pre/post with 6 items from the Patient Reported Outcome Measurement Information System [29]. Items were rated on a 5-point scale (1 = Poor, 5 = Excellent) and summed (higher scores indicate more positive perceptions). Scores were aggregated to compute a total score at each time point (α_Baseline_ = 0.86, α_3−months_ = 0.76) with higher scores indicating more positive perceptions of one’s health. *Satisfaction with the intervention* was measured at follow-up with the 8-item Client Satisfaction Questionnaire (CSQ) [30]. Items were rated on a 4-point scale and summed (higher scores indicate more satisfaction). Higher scores indicate more satisfaction with the intervention at the 3-month follow-up (α = 0.85). At follow-up, participants’ were queried on their *perceptions of the intervention* [31] using open-ended questions (e.g., which aspects of the peer sessions they liked/disliked, advantages/disadvantages of this approach relative to other healthcare services, if/how the sessions helped them achieve their health goals, preferred characteristics of the peer, and recommended changes). At the end of the study, staff and peers were also queried about their perceptions of the implementation potential of the intervention (e.g., ease of communications with the peer, if/how communications impacted the care provided to patients, how working with a peer could impact a patient’s health, and recommended changes).

### Analyses

Descriptive statistics quantified metrics for feasibility (rates of recruitment and retention and intervention fidelity) and acceptability (Means/SDs for peer sessions attended and CSQ scores). Based on prior studies of homeless patients participating in peer-based interventions, our a priori benchmark for engagement was an average of ≥ 6 sessions completed [32, 33]. For satisfaction, the 8 CSQ items are rated on a 4-point scale, and a response of ≥ 3 indicates at least some satisfaction. Therefore, a mean of 24 on the CSQ was our benchmark for satisfaction. For fidelity, benchmarks for activities from the intervention components of Whole Health Coaching, Discussions of Healthcare Utilization, and Provider Communications were ≥ 80% (“high fidelity) and ≤ 50% (“low fidelity”) of participants [34]; no benchmark was set for the Peer Support activities as they were dependent on participants’ needs. To evaluate utility, we calculated frequencies of acute care utilization (hospitalizations, ED visits) and mean/SDs for non-acute care utilization (primary care, homeless, mental health, and other medical services) in the pre/post period, and conducted paired samples t-tests to explore within-person changes in patient engagement and perceptions of health. The study was not powered to detect significant changes pre/post; thus, analyses were conducted to estimate the magnitude of change in outcomes using Cohen’s *d*.

Qualitative data were analyzed with rapid analysis [35]. Templated summaries of interview responses were entered into a matrix, with a domain name for each interview question placed on the vertical axis and participants listed on the horizontal axis. Two authors (DB, JW) independently reviewed discrete copies of the matrices to develop concise summaries to organize the data and identify global themes across the interview questions related to factors that hindered or facilitated participants satisfaction and engagement with the intervention. The authors then met to compare their summaries, engaged in a consensus process, and created a final matrix that reflected their agreed upon global themes.

## Results

### Intervention engagement and satisfaction

Of all 20 participants enrolled, 127 peer sessions were completed over three months (M = 6.35, SD = 3.5; Median = 7; Min/Max = 0–12). On average, peers reported 222 min (3.7 h) of direct contact per patient (SD = 192.3); the median number of minutes spent with patients was 173 (2.9 h). Thirteen patients (65%) met the benchmark for engagement in the peer sessions (i.e., ≥ 6 sessions) Due to the COVID-19 pandemic, almost all sessions (123 of 127) were conducted by telephone; three sessions were conducted in-person; one session by video. The average CSQ score was 28.75 (SD = 2.79), and 14 (out of 16) participants exceeded the a priori benchmark on this measure.

### Fidelity

**Fig. 2:**
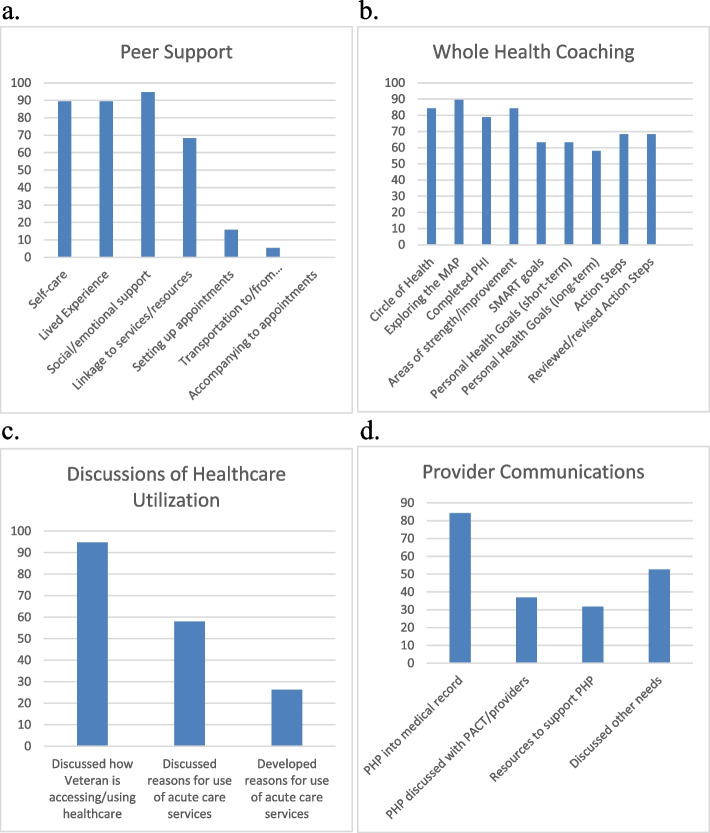
Percentage of participants for which peers reported engaging in intervention activities (**a**. peer support, **b**. Whole Health Coaching, **c**. Discussions of Health Care Utilization, **d**. Provider Communications)

Figure 2 provides of the percentage of participants for which peers reported engaging in specific intervention activities. The most common peer activities were discussions of self-care, sharing lived experiences, and providing social/emotional support (each conducted with ≥ 89% of participants). Assisting patients with setting up appointments, providing transportation to and from appointments, and accompanying patients to appointments were reported for a minority of participants. For health coaching, psychoeducational activities (e.g., reviewing the Circle of Health, exploring mission/aspiration/purpose) and completing the Personal Health Inventory were conducted with ≥ 78% of participants. Assisting patients with identifying personal health goals and action plans were conducted with the majority of participants (63% and 68%, respectively) but below the benchmark for high fidelity. Regarding discussions of healthcare utilization, peers discussed with almost all patients how they were accessing and using healthcare services. Discussions regarding reasons for using acute care services and developing strategies to minimize reliance on acute care were conducted with 58% and 26% of participants, respectively. For provider communications, entering Personal Health Plans into participants’ medical records and including providers as co-signers were conducted for over 84% of participants. Synchronous communications with providers to discuss these plans and how to support patients’ personal health goals was conducted with a minority of participants (37%).

### Within-person changes in patient engagement and perceptions of health

Scores on patient engagement increased pre-to-post, though not significantly and effect size estimates were small in magnitude. Perceptions of health increased significantly from pre-to-post, with effect sizes moderate in magnitude. Analyses at the item-level indicated that improvements were driven primarily by increases in one’s perceptions of their physical and social health (see Table 2).

**Table 2:** Within-person changes in patient engagement and perceptions of health

Variable	Pre-treatment		Post-treatment		Paired Samples t-test		
	Mean	Median	Mean	Median	Cohen’s *d*	*t* (*df*)	*P*
	(SD)	(Min, Max)	(SD)	(Min, Max)	(95% CI)		
Patient Engagement	22.73 (4.20)	22.00 (16, 29)	23.80 (3.03)	15.00 (19, 29)	-0.28 (-3.14, 1.01)	-1.01 (14)	0.29
Perceptions of Health	14.73 (5.57)	14.50 (7, 29)	17.13 (3.99)	16.00 (11, 27)	-0.46 (-4.68, -0.12)	-2.26 (14)	0.04
General Health	2.47 (0.99)	2.00 (1, 5)	2.53 (0.83)	3.00 (1, 4)	-0.10 (-0.46, 0.32)	-0.37 (14)	0.72
Quality of Life	2.67 (1.23)	2.50 (1, 5)	2.67 (1.05)	3.00 (1, 5)	0.00 (-0.51, 0.51)	0.00 (14)	1.00
Physical Health	2.07 (1.03)	2.00 (1, 4)	2.53 (0.83)	3.00 (1, 4)	-0.63 (-0.88, -0.05)	-2.43 (14)	0.03
Mental Health	2.67 (1.54)	2.00 (1, 5)	2.67 (1.11)	2.00 (1, 5)	0.00 (-0.73, 0.73)	0.00 (14)	1.00
Satisfaction with Social Activities/ Relationships	2.13 (1.06)	2.00 (1, 5)	3.13 (0.99)	3.00 (1, 5)	-0.80 (-1.69, -0.31)	-3.09 (14)	0.01
Carrying Usual Social Activities/Roles	2.73 (1.28)	3.00 (1, 5)	3.60 (1.06)	3.00 (2, 5)	-0.58 (-1.70, -0.03)	-2.23 (14)	0.04

### Acute care and non-acute care service utilization

In the 3-months pre/post enrollment, 45% (*n* = 9) and 15% (*n* = 3) of participants, respectively, were hospitalized. In both the pre/post periods, 45% (*n* = 9) of participants had an ED visit. In the pre-enrollment period, participants had, on average, 2.45 (SD = 1.75) primary care, 3.05 (SD = 3.98) homeless service, 5.25 (SD = 7.80) mental health, and 5.05 (SD = 4.86) other medical care visits. In the post-enrollment period, participants had, on average, 3.65 (SD = 4.30) primary care, 2.15 (SD = 4.05) homeless service, 5.45 (SD = 11.08) mental health, and 6.70 (SD = 5.81) other medical care visits.

### Facilitators and barriers to engagement and satisfaction: qualitative feedback from patients

Trust in the peers because of their veteran status was a facilitator of engagement and satisfaction, with participants reporting feeling “comfortable sharing information because he was a veteran,” [ID-1007] “we spoke the same language… we’re both veterans,” [ID-1004]. Relatedly, when queried regarding preferred characteristics of a peer, 13 out of 16 participants stated no preference in terms of gender, race/ethnicity, or age; the peer being a fellow veteran with similar life experiences is what mattered most. Collectively, these data suggest that peers’ expertise in health topics was not as relevant to participants as was the congruence of the participants’ and peers’ lived experiences. Participants valued the social/emotional support received (“[peer] was really able to listen” [ID-1017], “He asked about my goals; encouraged me to be healthier, to walk more” [ID-1016]). Participants also valued peers’ efforts to connect patients to resources and help them navigate VA and community-based care systems (“He helped find connections to a dietician” [ID-1001], “…got me connected with the Pulmonary Department” [ID-1008], “…got information on transportation and help with smoking” [ID-1011], “…got a referral to mental health” [ID-1012].

Modality of sessions was noted as a barrier to engagement and satisfaction, with some participants reporting that they “preferred in-person sessions” [ID-1001], “would have liked to meet the peer in person” [ID-1012], “meet in person at least once per month” [ID-1018]. Another barrier was the length and intensity of the intervention, which was viewed by many as insufficient (“I would have liked to meet more than once per week…. not everything I needed was taken care of” [ID-1003], “It could have been longer sessions” [ID-1019], “…wished the sessions were longer than three months” [ID-1020]).

### Facilitators and barriers to implementation: qualitative feedback from providers and peers

Staff viewed the peer role itself as a facilitator to implementation of the intervention (“Patients can better relate to peers.” “They identify with peers”). For example, staff noted that patients are more willing to share information with peers, which allows providers to have a “fuller picture of what the veteran is going through.” Another staff member noted that a peer who is able to effectively communicate with a patient and provide a consistent message across the care team “reinforces [provider recommendations] and can build trust.” Staff also highlighted the practical value of the peers in terms of being able to “spend more time with a patient”, “be a liaison”, “help with transportation.” Regarding barriers, staff reported a lack of knowledge about the scope of the peer role and how peers could support PACTs (“we could have utilized [peer] more but didn’t know how”). Staff reported that most of their communications with peers occurred asynchronously (e.g., notes in the medical chart; emails) and preferred earlier and more frequent communications. Consequently, staff reported modest changes in patients’ care based on input from peers. Staff also reported a need for more integration of peers into the PACTs (“Have peer come to speak at one of their monthly meetings,” “…make peers a part of the clinical neighborhood”).

The two peers also noted challenges to communicating with PACTs and a need for guidance about this process. They further highlighted the importance of coordinating their efforts with the PACT and other providers because of the complex health issues of most patients. Peers also reported that more time was needed to help patients develop personal health goals, as initial sessions were often devoted to rapport-building and linking patients to resources to address their healthcare needs. Finally, both peers highlighted challenges in discussing patients’ acute care use and concerns that such discussions could be perceived by patients as suggesting that use of these services was inappropriate.

## Discussion

This study introduced an innovative model for addressing the healthcare needs of high-risk, high-need homeless veterans and described preliminary findings regarding its feasibility and acceptability across multiple stakeholders and its utility. Despite the transiency of the target population and restrictions due to the COVID-19 pandemic, we successfully contacted over half of eligible patients, obtained interest in study participation from almost half of those contacted, and retained 80% of participants after three months. Ratings of satisfaction and level of engagement with peers suggested a high degree of acceptability of this intervention, which is notable given the delivery was almost entirely virtual. The ability to trust and relate to peers was a key driver of satisfaction and engagement, which is consistent with other research [26, 36]. Social support has previously been reported as a key benefit of peers, particularly for homeless adults who are often socially isolated [37]. Improvements in perceptions of health for study participants were primarily driven by social health items. The significance of this is underscored by the fact that improvements in social support have been linked to improvements in health problems for homeless adults [38]. Finally, significant improvements in perceptions of health, reduced hospitalizations, and increased engagement in supportive care services suggest the potential utility of this approach [39].

The findings highlighted some challenges to feasibility and acceptability. For example, patients reported the lack of in-person contact as a barrier to engagement and satisfaction. Although largely a function of pandemic-related restrictions in place during the study, virtual care will likely become more common for the delivery of all types of healthcare services in the future, particularly mental health care [40]. For many types of care, the effectiveness of delivery via virtual modalities is comparable to that of in-person delivery [41]. However, it is not clear if virtual care is effective for high-risk, high-need homeless patients as many wraparound interventions for this population include outreach to facilitate an understanding of the impact of a patient’s environment on their health [42, 43]. More research is needed to determine if, and under what circumstances, virtual care is effective for homeless adults [44]. The length and overall intensity of the intervention were also noted by patients and peers as a barrier to both satisfaction and implementation. Although designed to be an adjunct to primary care, there may be value in extending the intervention length to allow peers sufficient time to support the complex needs of patients as well as assist them with developing personal health goals. Extending the length of the intervention to six months, for example, would align Peer-WHC with similar peer-based interventions for vulnerable populations [45–47].

The current study extends a recent study on use of health coaching with high-risk patients [48] by testing this approach with homeless veterans and integrating the roles of a health coach with those of a peer specialist. Although complementary in many ways, health coaches use patient preferences to develop personal health goals, whereas peers use their lived experience to provide social-emotional support to patients. Assisting patients with developing personal health goals was feasible for peers in the study, which is consistent with other research on vulnerable adults [49, 50]. However, this role was less common than other health coaching activities that were tracked. This may have been attributable to the nature of the population in which acute health problems are common, thus requiring stabilization and resource linkage to be prioritized [51]. Extending the length of the intervention and prioritizing care navigation and linkage as an initial step prior to assisting patients with developing personal health goals may facilitate this process and better align the approach with the needs of the population. Other challenges reported by peers such as discussing patients’ use of acute care and communicating with PACTs suggest a need for further training of peers in these activities. Lack of knowledge of the peer role on the part of providers is a common barrier to integration of peers into primary care [52]. Education of primary care staff may be critical to successful implementation of Peer-WHC in these settings.

In terms of limitations, because this was a single-arm trial, the effectiveness of Peer-WHC to reduce acute care utilization or improve health outcomes cannot be determined from this study. In addition, although significant changes were observed for some self-reported outcomes and changes in acute and non-acute care utilization were in the expected directions, statistical power was limited by the small sample size. Generalizability of the current findings are also limited by the homogeneity of the sample demographics. The findings may also not generalize to more chronically homeless patients, given that 90% of the sample reported stable housing at the time of enrollment. Future studies that are adequately powered and enriched with a more diverse sample of veterans, including those with unstable housing, will be needed to test the effectiveness of the intervention for improving health outcomes.

In summary, Peer-WHC is novel intervention to address the care needs for homeless super-utilizer veterans. The findings from the current trial support the feasibility, acceptability, and utility of this approach for homeless veterans. Further, the intervention may be improved by additional time for the peer relationship to foster the patient’s identification and development of personal health goals, and building more collaborative relationships between the peer and the patient’s healthcare providers. A future randomized controlled trial is warranted to test the impact of the intervention on reducing these patients frequent use of acute care services while maintaining quality of care and minimizing costs.

## Data Availability

The datasets generated and analyzed during the current study are not publicly available, but are available from the corresponding author on reasonable request.

## Abbreviations

CSQ: Client Satisfaction Questionnaire
NCHAV: National Center for Homelessness Among Veterans
PACT: Patient Aligned Care Teams
VHA: Veterans Health Administration
WHC: Whole Health Coach/Whole Health Coaching
